# Metformin Attenuates Brain Injury by Inhibiting Inflammation and Regulating Tight Junction Proteins in Septic Rats

**DOI:** 10.22074/cellj.2020.7046

**Published:** 2020-09-08

**Authors:** Fatima Ismail Hassan, Tina Didari, Maryam Baeeri, Mahdi Gholami, Hamed Haghi-Aminjan, Madiha Khalid, Mona Navaei-Nigjeh, Mahban Rahimifard, Sara Solgi, Mohammad Abdollahi, Mojtaba Mojtahedzadeh

**Affiliations:** 1. Pharmaceutical Sciences Research Center (PSRC), The Institute of Pharmaceutical Sciences (TIPS), Tehran University of Medical Sciences, Tehran, Iran; 2. School of Pharmacy, Tehran University of Medical Sciences, Tehran, Iran; 3. Pharmaceutical Sciences Research Center, Ardabil University of Medical Sciences, Ardabil, Iran

**Keywords:** Brain Injury, Metformin, Molecular Mechanisms, Sepsis

## Abstract

**Objective:**

Metformin has a potent inhibitory activity against inflammation and oxidative stress, which inevitably occur in sepsis-associated encephalopathy (SAE). The precise mechanisms underlying neuroprotective effects of metformin in SAE, are still unclear. In the present work, the protective effect of metformin on SAE using cecal ligation and puncture (CLP) model of sepsis, was assessed.

**Materials and Methods:**

In this experimental study, CLP procedure was performed in Wistar rats and 50 mg/kg metformin was administered immediately. Specific markers of sepsis severity, inflammation, blood brain barrier (BBB) dysfunction, and brain injury, were investigated. Specific assay kits and real-time polymerase chain reaction (RT-PCR) were used. Histopathological assessment was also carried out.

**Results:**

Treatment with metformin decreased murine sepsis score (MSS), lactate, platelet lymphocyte ratio (PLR), and high mobility group box (HMGB1) levels. The expression levels of claudin 3 (Cldn3) and claudin 5 (Cldn5) were increased following treatment with metformin. Metformin decreased the expression of S100b, neuron specific enolase (Nse), and glial fibrillary acidic protein (Gfap).

**Conclusion:**

Our study suggests that metformin may inhibit inflammation and increase tight junction protein expressions which may improve BBB function and attenuate CLP-induced brain injury. Hence, the potential beneficial effects of metformin in sepsis, should be considered in future.

## Introduction

Sepsis remains the most common disease among the critically ill with no specific diagnosis and treatment. Sepsis-associated encephalopathy (SAE) is the common form of delirium observed in septic patients (1). It affects the blood-brain barrier (BBB) function and other brain cells as a result of dysregulation of cytokines and neurotransmitters production (2). SAE involves the release of inflammatory mediators, oxidative stress induction and increase in other biomarkers that damage brain cells and affect BBB integrity and intracellular metabolism (2, 3). Neuroinflammation, ischemic processes, and neurotransmitter dysfunction are processes involved in the pathophysiology of SAE (4). Previous studies reported that microglial and astrocytes activation affect BBB integrity and stimulate the production of several mediators in the brain as observed in SAE (4, 5). Inflammation and oxidative stress are crucial in SAE and can lead to other detrimental effects.

Metformin was shown to exert its protective role in sepsis partly through its anti-inflammatory and antioxidant properties (6). Neuroprotective effects of metformin were reported to be mediated via these mechanisms. Metformin prevented brain mitochondrial dysfunction by reducing oxidative stress levels in high fat diet-induced insulin resistant rats, promoted neurogenesis and improved spatial memory, protected against cerebral ischemia, and enhanced angiogenesis in post-stroke recovery (7- 10). Several studies reported the protective effects of metformin in sepsis (11). Metformin was shown to inhibit pro-inflammatory cytokines production, down-regulate myeloperoxidase expression, and decrease creatine kinase myocardial band, and brain natriuretic peptide in endotoxin-induced myocarditis (12). It protected the lung of septic rats against neutrophil infiltration, inflammation, and oxidative damage (13). Metformin attenuated sepsis-induced brain injury by inhibiting oxidative stress and decreasing BBB permeability via activating phosphatidylinositol-3 Akt signaling pathway (14). However, the exact mechanism by which metformin exerts its neuroprotective effects is not clear yet. Elucidation of other SAE markers and pathways is necessary.

In this study, the neuroprotective effects of metformin on SAE were investigated, using cecal ligation and puncture (CLP) model. We hypothesized that using CLP model, metformin will improve sepsis severity, restore BBB function, and attenuate brain injury by reducing the levels of inflammatory markers, increasing gene expression of tight junction proteins, and decreasing the gene expression of brain injury markers which will further reduce brain structural damage.

## Materials and Methods

In this experimental study, Metformin (>97% purity) was purchased from (Soha Pharmaceutical Company, Iran). Ketamine and xylazine used for anesthesia induction, were purchased from Alfasan, Netherlands, and normal saline was bought from B-braun, Germany. Formalin, rat high-mobility group protein B1 (HMGB1) ELISA and lactate assay kits were obtained from ZellBio GmbH (Germany). RNase solution, iScript cDNA synthesis kit and propidium iodide were obtained from Sigma-Aldrich (Germany).

### Experimental design

All protocols and procedures regarding animal handling were approved by Tehran University of Medical Sciences Ethics Committee under the reference number IR.TUMS. VCR.1396.2075. In this experimental study, 36 healthy adult male Wistar rats (200-250 g) aged 8-10 weeks, were obtained from the animal facility of Pharmacy, Tehran University of Medical Sciences (TUMS). All rats were kept at 25 ± 1°C, with 50% humidity and 12-hour day/ night cycle, and had access to standard feed and water ad libitum. Six (6) rats were randomly assigned to each group. Group I: Sham (12 hours), group II: CLP (12 hours), group III: CLP+metformin 50 mg/kg (12 hours), group IV: Sham (24 hours), group V: CLP (24 hours), and group VI: CLP rats administered metformin 50 mg/ kg (24 hours). Rats were sacrificed after 12 and 24 hours, depending on the grouping.

### Cecal ligation and puncture model

CLP was performed to induce sepsis in rats as previously described (14). Rats were anesthetized with intraperitoneal ketamine/xylazine (110/10 mg/kg body weight). The lower quadrant of the abdomen was shaved and disinfected and then, a longitudinal skin midline incision was made. The mesentery of the cecum was carefully dissected and the cecum was ligated at the designated position for sepsis induction. The cecum was perforated using 18-gauge needle and two punctures. After removing the needle, a small amount (droplet) of feces, was extruded. The cecum was carefully returned into the abdominal cavity. Prewarmed normal saline (37°C, 5 ml per 100 g body weight) was administered subcutaneously to resuscitate the animals and then, the animals were placed back in cages in a temperature-controlled room (22°C) with 12-hour light and dark cycles and free access to water and food. All above procedures were performed on sham animals except cecum ligation and puncture.

### Murine sepsis score

This scoring was used to determine disease severity and mortality as previously described (15). The method has been successfully carried out in rats (16). This scoring system involves specific variables and numbers that indicate sepsis severity. The score ranged 0 to 4 and involved appearance, level of consciousness, activity, response to stimuli, eyes, respiration rate and quality. Two investigators scored the animals 12 and 24 hours after sepsis induction where one of the investigators was blinded to treatment.

### Blood and organ sampling

Blood samples and brains were taken for determination of platelet lymphocyte ratio (PLR), and levels of lactate, high-mobility group box one (HMGB1), genes expression, and histopathological evaluations. Arterial blood (3 ml) was collected from the left arteria carotis into plain and heparinized tubes. The samples in the heparinized bottles were kept for complete blood count analysis while those in plain bottles were centrifuged at 3000 g for 15 minutes. The plasma was separated, kept in different tubes, and stored at -80˚C for further analysis. Brain samples were washed with normal saline and divided into two hemispheres. The first was stored at -8˚C for real-time polymerase chain reaction (RT-PCR) analysis, while the second part was stored in 10% formalin for histopathological studies.

### Platelet lymphocyte ratio determination and lactate measurement

PLR determination is used to evaluate inflammatory responses (17) while lactate is an important marker of sepsis severity. PLR was determined from the complete blood count measured using an automated analyzer (Sysmex, America Inc.). Platelet counts obtained were divided by lymphocyte counts for each sample to obtain the platelet/lymphocyte ratio. We measured plasma lactate concentration using a lactate assay kit (ZellBio GmbH, Germany) according to the manufacturer’s instruction and calculations were done using the standard formula.

### HMGB1 measurement

HMGB1 is an important marker of late sepsis lethality. It was used to evaluate the effect of metformin in late sepsis. HMGB1 measurement was performed in the rat’s brain that was stored at -80˚C The brain sample was homogenized with phosphate buffer and centrifuged. Specific rat ELISA kit (ZellBio GmbH, Germany) was used according to manufacturer’s protocol and HMGB1 level was calculated using standard curve.

### Gene expression evaluation using real-time polymerase chain reaction

The expression level of gene of the specific markers for BBB integrity and brain injury were evaluated (4, 5, 13). These included claudins 3 (*Cldn3*), claudin 5 (*Cldn5*), *S100b*, neuron specific enolase (*Nse*), and glial fibrillary acidic protein (*Gfap*). Total RNA was extracted from brain samples stored at -80oC using TRIzol® reagent according to the manufacturer’s protocol. The genomic DNA was extracted using DNase I, RNase-free kit (Fermentas, Glen Burnie, MD, USA). The concentrations of RNA and DNA were determined by Thermo Scientific NanoDrop 2000c UV-Vis spectrophotometer (Thermo Scientific, USA). Complementary DNA (cDNA) was then reverse transcribed using the Thermo Scientific RevertAid First Strand cDNA synthesis kit as per manufacturer’s manual. For analysis of genes expression levels, primer pairs were used with glyceraldehyde-3-phosphate dehydrogenase (*Gapdh*) as the housekeeping gene. Real time RT-PCR, to determine the expression level of *Cldn3*, *Cldn5*, *S100b*, *Nse*, and *Gfap*, was performed on LightCycler® 96 System (Roche) using the SYBR Green master mix. Expression analysis for each sample was done in triplicates. Cycle number of each reaction was calculated from the amplification curve to determine the relative gene expression using the comparative cycle threshold method. The double delta Ct (2^-ΔΔct^) analysis was done to determine the differences in gene expressions among the groups (18). The primer sequences used for RT-PCR in terms of genes as follows:

*Cldn5*-

F: 5ˊ-CTTGTGAGGACTTGACCGA-3ˊ

R: 5ˊ-GTAGGAACTGTTAGCGGCA-3ˊ

*S100b*-

F: 5ˊ-GTATAGCACTGGTTGTAGAC-3ˊ

R: 5ˊ-CAGCATACATTACACCTAAGA-3ˊ

*Gfap*-

F: 5ˊ-CCAACTAACAGGATACTCAC-3ˊ

R:5ˊ-ATAACAACAAGGATGAAGGAA-3ˊ

*Nse*-

F: 5ˊ-GTCGTCTGCCATTACTCTAC-3ˊ

R: 5ˊ-ACCATTGCTAACCTTTCTGT-3ˊ

*Cldn3*-

F: 5ˊ-ACCAAGATCCTCTATTCCG-3ˊ

R: 5ˊ-TACATCGACGGTTGGTAG-3ˊ

*Gapdh*-

F: 5ˊ-ACTGAGCAAGAGAGGCCCTA-3ˊ

R: 5ˊ-TATGGGGGTCTGGGATGGAA-3ˊ

### Histopathological studies

The harvested tissue (brain) was fixed in 10% neutral buffered formalin (NBF, pH=7.26) for 48 hours, then processed and embedded in paraffin. The 5-μm thick sections were prepared and stained with haemtoxylin and eosin (H&E). Two sections per animal in each group were examined. An independent (blinded) pathologist performed histopathological analysis using light microscopy (Olympus BX51, Olympus, Japan). Histopathological changes, including acute and chronic inflammatory response, liquefactive necrosis, hemorrhage and/or hyperemia in different samples, were evaluated.

### Statistical analysis

In the present work, we present our results as mean ± standard deviation (± SD) or as median and interquartile range (25 and 75% quartile). One-way analysis of variance (ANOVA) or Kruskal Wallis’s ANOVA followed by Tukey or Dunn’s post hoc tests for multiple comparisons, was performed. Pearson correlation measurement was also performed. Significance was considered at P≤0.05 among the groups. (Stats Version 3.2.10).

## Results

### Murine sepsis score was lower in metformin-treated cecal ligation and puncture rats

**Fig.1:**
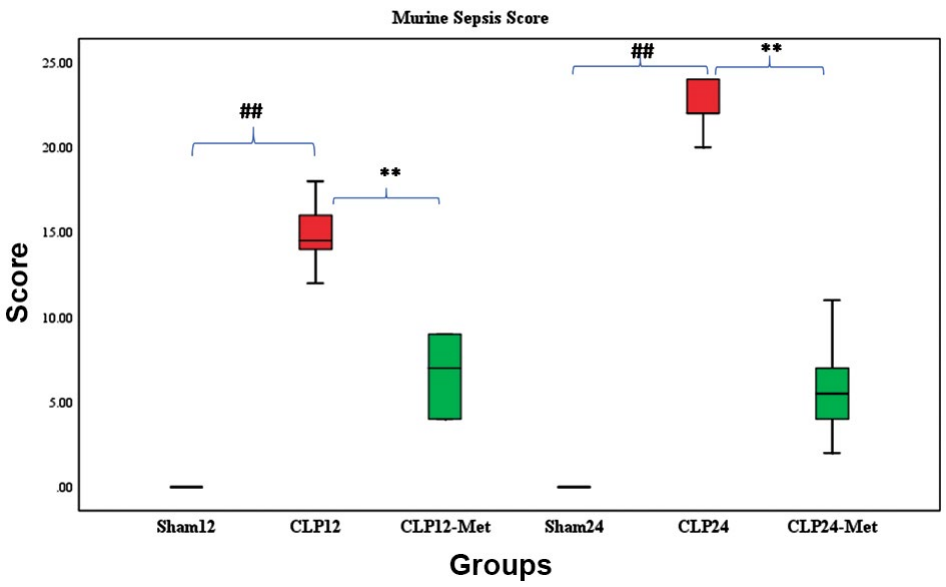
Effect of metformin administration on sepsis severity. Following treatment with metformin 50 mg/kg, sepsis severity was reduced 12 and 24 hours post CLP, as determined by a murine sepsis scoring method. Statistical evaluation of results using Kruskal Wallis’s ANOVA followed by Dunn’s post hoc revealed a significant difference among the groups for MSS (P<0.001). Values are median and interquartile range. A significant difference was observed between Sham and CLP groups, and between CLP and metformin-treated groups. CLP; Cecal ligation and puncture, ##; P<0.01 for Sham12 versus CLP12, **; P<0.01 for CLP12 versus CLP12-Met, ##; P<0.01 for Sham24 versus CLP24, and **; P<0.01 for CLP24 versus CLP24-Met, n=6.

Figure 1 shows the effect of metformin administration on sepsis severity. MSS was used to determine disease progression in CLP rats. MSS was considered the sum of scores of all clinical variables for each rat. The interrater reliability was excellent (0.97) when calculated using the intra class coefficient (ICC). The average total score was taken to reconcile differences between scorers. The coat of animals in the sham groups (12 and 24 hours) remained smooth, and activity, response to touch, auditory stimuli, posture, respiration rate and quality were also normal. In CLP 12-hour group, patches of hair were piloerected, activity was slow, strong response to touch was observed, and respiration rate and quality were moderately reduced and labored, respectively. In the metformin-treated group (12 hours), patches of hair were mildly piloerected, rats were active but avoided standing upright, activity was slightly suppressed but response to touch was immediate, the eyes were not fully open but respiration rate and quality were normal. In the CLP 24 hour group, rats appeared puffy, activity was impaired and they remained stationary, there was no response to auditory stimuli, eyes were half open with secretions, respiratory rate and quality were severely reduced and labored, respectively. However, in the metformintreated groups (both 12 and 24 hours), the appearance was normal, rats were active but avoided standing upright, response to auditory stimulus was immediate, eyes were half open, respiratory rate and quality were normal but slightly labored.

Statistically significant differences (P<0.001) was observed when sham groups were compared with CLP groups at both 12 and 24 hours. Significant difference (P<0.001) was also observed when CLP groups were compared with metformin-treated groups at both 12 and 24 hours (Fig .1).

### Metformin decreased lactate and HMGB1 levels and platelet lymphocyte ratio following cecal ligation and puncture-induced sepsis

**Fig.2:**
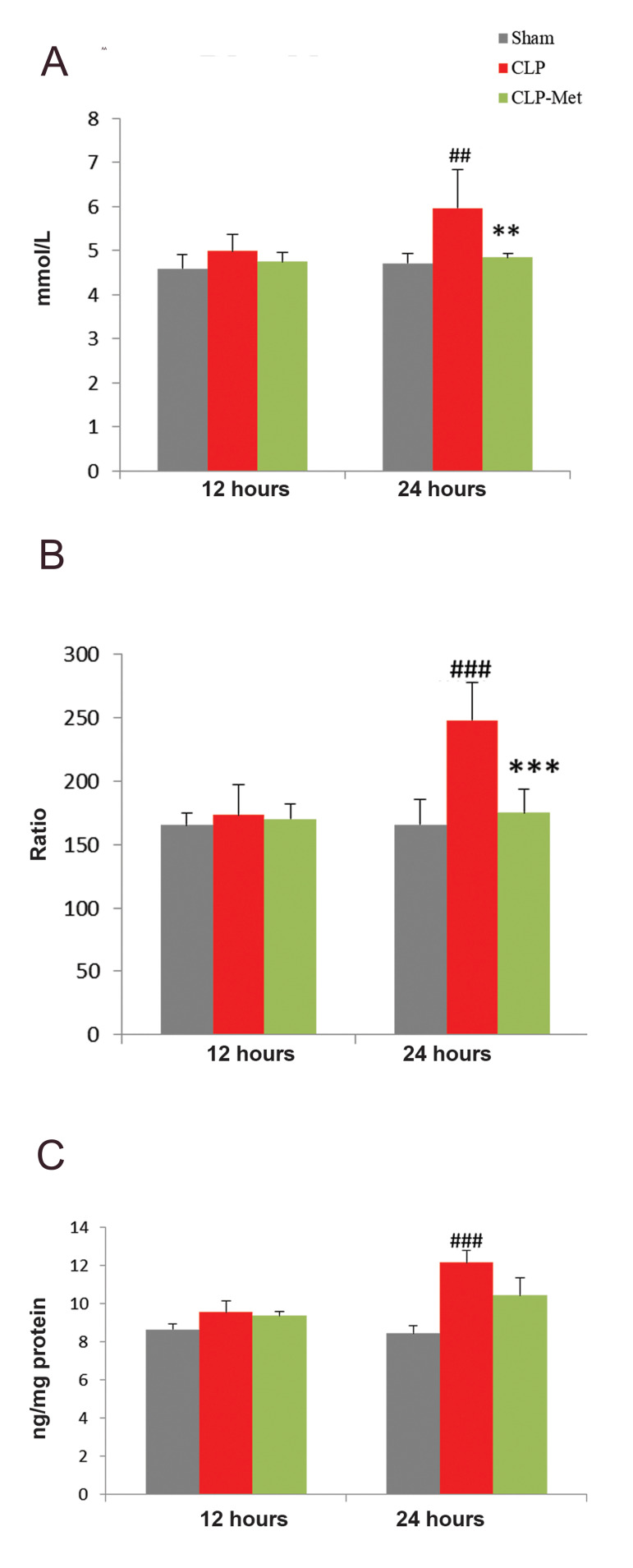
Effect of metformin administration on blood lactate and HMGB1 levels and PLR. **A.** CLP increased blood lactate, **B.** PLR, and **C.** HMGB1 in rats. Values are expressed as mean ± SD, n= 6. Statistical evaluation of results using oneway ANOVA revealed a significant difference among the groups for blood lactate levels (P<0.001), PLR (P<0.001), HMGB1 (P<0.001). PLR; Platelet lymphocyte ratio, CLP; Cecal ligation and puncture, ##; P<0.01, ###; P<0.001 as CLP compared with sham, **; P<0.01, and ***; P<0.001 between metformin-treated group and the CLP.

Figure 2 presents the changes associated with blood lactate, PLR, and HMGB1 after metformin administration, measured 12- and 24-hour post CLP. No statistically significant difference was observed among the groups after 12 hours in blood lactate (Fig .2A). However, 24 hours post CLP, blood lactate significantly increased when compared with sham (P<0.01). Metformin 50 mg/ kg significantly (P<0.01) decreased blood lactate. The PLR was calculated from platelets and lymphocytes count taken 12 and 24 hours after CLP. The PLR values obtained were similar between sham12, CLP12, and CLP-Met12 groups, but 24-hour post CLP, a significant (P<0.001) increase in CLP24 group compared with sham24 was observed. A significant (P<0.001) decrease in metformintreated group (CLP-Met24) compared with CLP24 group, was also observed (Fig .2B). No significant difference in HMGB1 concentration was observed among the groups. After 24 hours, a significant (P<0.001) increase was observed in CLP group compared with sham (Fig .2C). Metformin treatment did not significantly decrease HMGB1 concentration in CLP rats.

### Metformin improved blood brain barrier function following cecal ligation and puncture-induced sepsis

**Fig.3:**
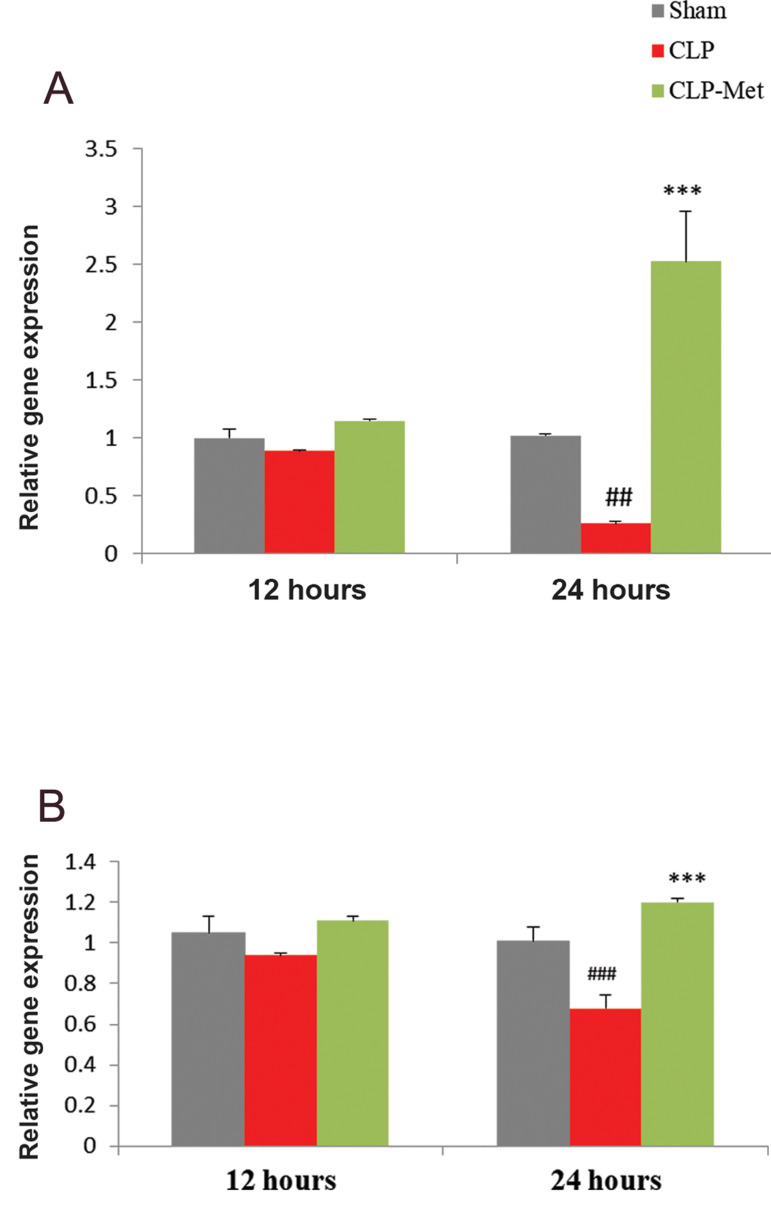
Effect of metformin administration on *Cldn3* and *Cldn5* gene expressions. CLP decreased A. *Cldn3* and B. *Cldn5* expression in rats. Values are expressed as mean ± SD. Statistical evaluation of results using one-way ANOVA, revealed a significant difference between groups for *Cldn3* (P<0.001), and *Cldn5* (P<0.001). CLP; Cecal ligation and puncture, ##; P<0.01, ###; P<0.001 as CLP compared with sham, and ***; P<0.001 between metformin-treated group and CLP.

Figure 3 presents the expression of tight junction proteins after metformin treatment using real time RTPCR. Metformin increased the expression of claudin 3 and 5 following a reduction induced by sepsis. No statistically significant difference was observed in the expressions of both *Cldn3* (Fig .3A) and *Cldn5 *(Fig .3B) 12 hours post CLP. However, 24 hours post CLP, a significant decrease (P<0.01) was observed in *Cldn3* expression when sham was compared to CLP, but a significant increase was observed when CLP was compared with metformin treated group. For *Cldn5* expression, a significant difference (P<0.001) was observed when sham was compared with CLP group, and a significant (P<0.001) increase in metformintreated group when compared with CLP group 24 hours post CLP.

### Metformin attenuated brain injury following cecal ligation and puncture-induced sepsis

**Fig.4:**
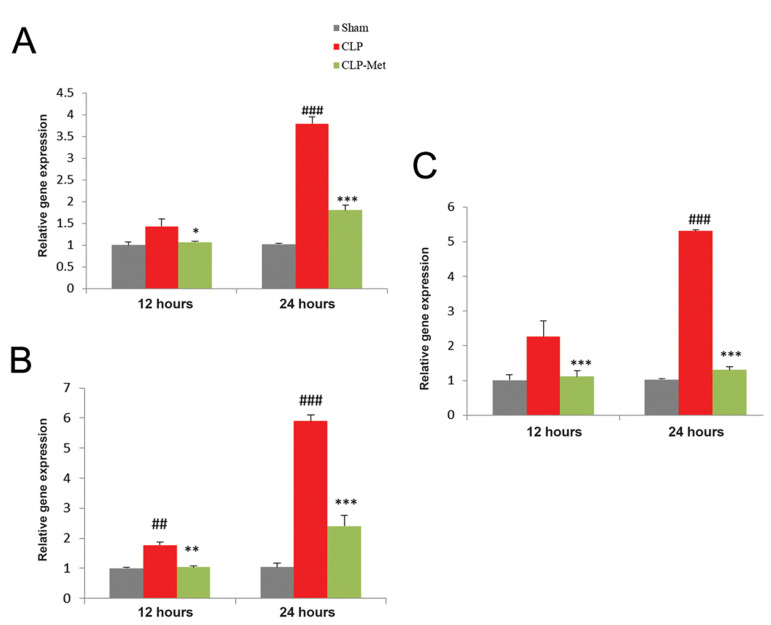
Effect of metformin administration on *S100b*, *Nse*, and *Gfap* gene expressions. **A.** S1006, **B.** *Nse* and **C.** Gfap. Values are expressed as mean ± SD, (n=6). Statistical evaluation of results using one-way ANOVA revealed a significant difference between groups for *S100b* (P<0.001), *Nse* (P<0.001), and *Gfap* (P<0.001). CLP; Cecal ligation and puncture, ##; P<0.01, ###; P<0.001 between CLP and sham, *; P<0.05, **; P<0.01, and ***; P<0.001 between metformintreated group and the CLP.

Figure 4 presents changes in specific brain injury markers after metformin treatment. The expression levels of *S100b*, *Nse*, and *Gfap* were determined using real time RT-PCR. The expression of *S100b* (Fig .4A) was significantly increased (P<0.01 and P<0.001) in CLP (12 and 24 hours) compared to sham (12 and 24 hours) groups respectively. A significant (P<0.05 and P<0.001) decrease was observed in CLP groups compared to metformin-treated groups 12- and 24- hour post CLP, respectively. The expression of *Nse* was significantly increased (P<0.01 and P<0.001) in CLP groups compared to sham and significantly decreased (P<0.01 and P<0.001) in metformin-treated groups compared to CLP groups 12 and 24 hours after CLP (Fig .4B). The expression of *Gfap* was significantly increased (P<0.001) in CLP groups compared to sham 12 and 24 hours (Fig .4C). Metformin significantly decreased (P<0.001) the expression of *Gfap* compared to CLP groups 12 and 24 hours post CLP.

### Metformin improved brain damage caused by the cecal ligation and puncture-induced sepsis

**Fig.5:**
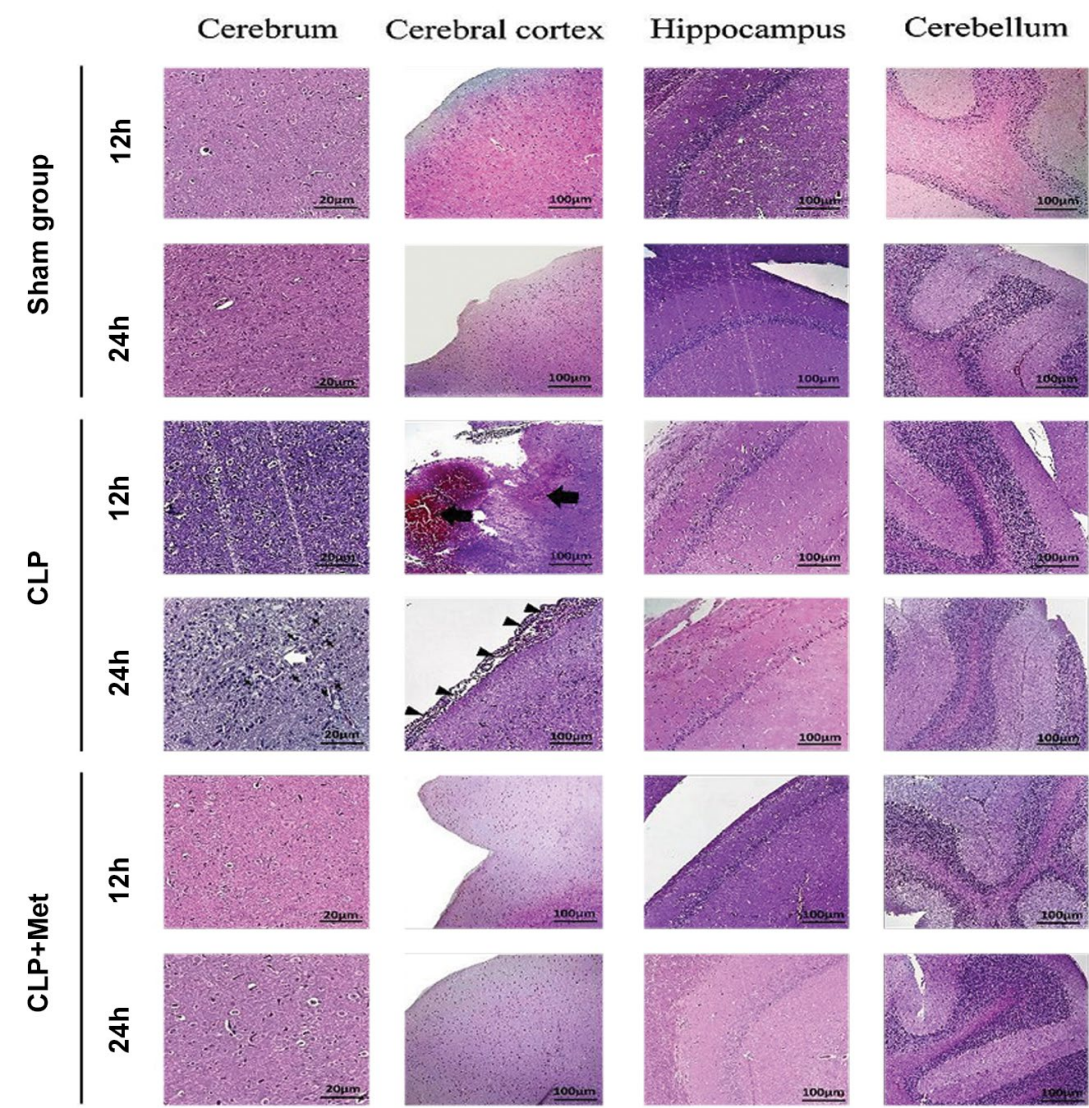
Effect of metformin administration on the morphology of the brain in cecal ligation and puncture (CLP)-induced sepsis. Cerebellum, cerebrum, cerebral cortex, and the hippocampus stained with H&E demonstrating cerebral hemorrhage, necrosis, and infiltration of inflammatory cells. Black thick arrows, white arrows, and thin arrows indicate cerebral hemorrhage, accumulation of inflammatory cells, and infiltration of inflammatory cells (meningitis), respectively. Magnification for cerebrum is ×400 while that of cerebral cortex, hippocampus, and cerebellum is ×200 [scale bars: 20 μm (cerebrum), and 100 μm (cerebral cortex, hippocampus, and cerebellum].

All H&E-stained brain sections from different experimental groups were evaluated histologically. The histopathological micrographs of brain sections in sham group were normal without any histopathological changes. Cerebral hemorrhage and meningitis were observed in the cerebrum 12 hours after CLP. In the 24 hour post-CLP group, meningitis, cerebral necrosis, and infiltration of inflammatory cells were observed. Micrographs of the brain sample in the metformin-treated groups both 12 and 24-hour post CLP, showed normal hippocampus, cerebral cortex, and cerebellum (Fig .5).

### Correlations

Correlation analysis between MSS and lactate as markers of sepsis severity, and PLR, and HMGB1 as inflammatory markers; and *Cldn3*, *Cldn5*, *S100b*, *Nse*, and *Gfap* as brain injury markers, are shown in Table 1. By using the Pearson correlation, it was observed that a statistically significant correlation exists between lactate and inflammatory markers (r=0.983, 0.914, P<0.01, P<0.001) and lactate and brain injury markers (r=0.975, 0.974, 0.992, P<0.001, P<0.001). A negative correlation with *Cldn5* (r=-0.868, P<0.05) was observed, while none was observed with *Cldn3* (r=-0.518). A statistically significant correlation was observed between MSS and lactate, inflammatory, and brain injury markers except for claudin 3 and 5 (r=0.928, 0.812, P<0.05, P<0.01).

**Table 1:** Correlation analysis for murine sepsis score (MSS), lactate, inflammatory and brain injury markers


Parameters	Pearson correlation (r)	Lactate (mmol/L)	MSS

Lactate (mmol/L)	R	1	0.928^##^
	P value		0.01
MSS	R	0.908^#^	1
	P value	0.05	
PLR	R	0.983^###^	0.812^#^
	P value	0.001	0.050
HMGB1	R	0.914^#^	0.812^#^
	P value	0.05	0.050
Cldn3	R	-0.465	-0.518
	P value	0.353	0.292
Cldn5	R	-0.868^#^	-0.522
	P value	0.05	0.288
S100b	R	0.975^##^	0.812^#^
	P value	0.001	0.050
Nse	R	0.974^##^	0.812^#^
	P value	0.001	0.050
Gfap	R	0.992^###^	0.928^##^
	P value	0.001	0.01


#, ##, and ###; Pearson correlation is significant at the level of P<0.05, P<0.01, and P<0.001 (2 tailed), respectively.

## Discussion

The present work investigated the effects of metformin on SAE using the CLP sepsis model. The results showed that CLP significantly increased murine sepsis score, and induced inflammation enough for disrupting the BBB and ultimately causing brain injury because of the release of various inflammatory and brain injury biomarkers. However, treatment with metformin 50 mg/kg reduced the inflammation, and improved brain damage, and BBB function thereby attenuating brain injury.

Microcirculatory failure, endothelial activation, BBB disruption, neuroinflammation, and altered brain signaling are complex mechanisms involved in SAE (19). Brain signaling is important in detecting the presence of chemicals released by microorganisms. Circumventricular organs and the vagus nerve are the pathways involved in neuroimmune communications where systemic inflammation is detected via toll-like receptors, CD14, and cytokine receptors, and visceral inflammation via axonal cytokine receptors (20). Neurotoxic substances such as nitric oxide, reactive oxygen species (ROS), cytokines, and glutamate result in cell death within the brain, which is a consequence of cytokine-induced microglial activation (21). Inflammatory cytokines such as tumor necrosis factor-alpha (TNF-α), interleukins, and HMGB1 are crucial in endothelial damage, BBB dysfunction, neuronal damage, and brain cell death (22, 23). In sepsis, microglial and astrocyte activation was linked with the release of inflammatory cytokines, including TNF-α and HMGB1 (23, 24). The BBB prevents neurotoxic substances such as cytokines and ROS from reaching the brain and its breakdown is associated with brain injury and edema which are common features of SAE (25).

The BBB consists of important tight junction proteins present in endothelial cells that regulate the movement of substances. In neurological diseases, the concentration of these proteins is dramatically reduced and this leads to BBB disruption (26). The consequences of BBB disruption involve the passage of neurotoxic substances which interact with brain cells and cause brain injury. Specific proteins in the brain cells such as S100B and GFAP, are released into plasma or cerebrospinal fluid (CSF) during brain injury (27).

CLP model is a gold standard in sepsis research, and it is resulted from polymicrobial infectious origin and closely related to human sepsis (14). Therefore, the beneficial effects of metformin in sepsis may give the researchers insight on how it may work in humans by inhibiting or preventing the deleterious responses associated with the disease.

Metformin was shown to be protective in sepsis. It was reported that metformin is effective in different models of sepsis mainly by inhibiting inflammation, oxidative stress, and apoptosis (12, 13). HMGB1 is implicated in infectious diseases during inflammatory response and tissue damage (28). In sepsis, increased circulating HMGB1 was shown to be associated with apoptosis and decreased survival rate. It is released in response to infection as an inflammatory cytokine and further stimulates an inflammatory response with subsequent tissue damage (29). HMGB1 was shown to cause microglial activation and cognitive dysfunction in lipopolysaccharide (LPS)-treated mice (22). Platelets accumulation was shown to result in organ failure through causing excessive inflammation, disseminated intravascular coagulation (DIC), and microthrombosis in sepsis (30). Platelets initiate inflammation by recruiting neutrophils and other cells to the site of infection or injury (31). This has made platelets important in detecting inflammation in sepsis. PLR is a new and cheap marker of inflammatory response (17). It has been used to detect the early onset of sepsis (32). The inhibitory effects of metformin on inflammation in lipopolysaccharide-treated cells and endotoxemia in mice were shown to be induced by inhibiting the release of HMGB1 a prominent proinflammatory cytokine and damage associated molecular pattern (DAMP) (33). In the present study, we found that metformin treatment inhibited inflammatory response by decreasing PLR and HMGB1 levels.

Hyperlactatemia is an important marker of sepsis severity and mortality. Decreased lactate clearance during sepsis was reported as a major cause of hyperlactatemia (28). Sepsis is believed to cause lactate accumulation by affecting pyruvate dehydrogenase (PDH), an enzyme responsible for the conversion of lactate to pyruvate. A human study reported significant declines in PDH during sepsis that was believed to be responsible for lactate accumulation (34). A distinctive feature of sepsis is impaired microcirculation with concomitant increase in lactate level (35). In the present study, we found that in CLP rats, blood lactate levels significantly increased but reduced by metformin administration. Since hyperlactatemia in sepsis depicts sepsis severity and mortality, the decreased MSS scores observed in metformin-treated groups compared to CLP, may be an indication of metformin’s ability to improve sepsis outcome. MSS was shown to exhibit high specificity in predicting sepsis severity and mortality (15).

Furthermore, BBB breakdown and brain injury are important features of SAE which occur as a result of the release of inflammatory mediators and ROS (25). Sepsis was shown to significantly alter the levels of tight junction proteins which are important components of the BBB (14, 36). Agents with antiinflammatory and anti-oxidant properties have been shown to preserve BBB integrity during sepsis by increasing the concentrations of these proteins (36). Activation of the microglial and astrocytes associated with cytokine production, affects the BBB since they help in maintaining its integrity in conjunction with TJ and transport proteins (22, 24). Metformin attenuated brain injury in CLP mice by inhibiting the inflammatory response, oxidative stress, and apoptosis (13). In our study, the concentration of PLR and HMGB1 (inflammatory markers) was decreased by metformin administration. The expression of *Cldn3* and *Cldn5* decreased significantly after CLP. Metformin treatment improved BBB function as evidenced by the significant increases in *Cldn3* and *Cldn5* expression.

In sepsis, BBB impairment results in the passage of neurotoxic substances which can cause brain injury associated with the release of several markers that damage the astrocytes and neurons (27, 37, 38). The presence of S100B, NSE, and GFAP in the serum indicates a loss in the BBB integrity as they are almost completely produced in the brain (28). An elevated level of these proteins is associated with cytokine activation in sepsis (19, 37, 38). S100B, NSE, and GFAP have been used to diagnose sepsis-induced brain injury (39). Metformin through its anti-inflammatory effects, may prevent BBB breakdown and subsequent brain injury. In the present study, the expressions of *S100b*, *Nse*, and *Gfap* were significantly increased in CLP groups which indicated sepsis-induced brain injury. Metformin treatment decreased these expressions significantly. In this study, obtained results indicated that metformin may improve BBB function and attenuate brain injury, as evidenced by decreased inflammatory markers, increased levels of tight junction proteins, and decreased levels of brain injury markers, and brain damage.

The histopathological studies revealed the presence of cerebral hemorrhage, meningitis, and cerebral necrosis and infiltration of inflammatory cells in the CLP groups, which were abolished in metformin-treated groups. The results of histopathological studies confirmed the inhibitory effects of metformin on inflammatory and brain injury markers release.

The correlation data revealed a strong positive correlation between markers of sepsis severity (MSS and lactate) and inflammatory and brain injury markers but a negative correlation with *Cldn5*. The effectiveness of metformin administration was evidenced by its ability to decrease these markers (sepsis severity, inflammatory, and brain injury) and increase *Cldn3* and *Cldn5* expressions.

## Conclusion

Metformin improved sepsis severity, and BBB function, and attenuated brain injury by decreasing sepsis score, lactate levels, inflammatory markers, and expression of brain injury markers, and increasing the expression of tight junction proteins. This study provides evidence on the potential therapeutic effects of metformin in SAE. As far as the authors are concerned, this study is the first document to report PLR and the neuroprotective effects of metformin that were mediated via the inhibition of *S100b*, *Nse*, and *Gfap* expression in a rat model of CLPinduced sepsis.
